# Transcriptional control of enterohepatic lipid regulatory targets in response to early cholesterol and phytosterol exposure in apoE^−/−^ mice

**DOI:** 10.1186/s13104-017-2859-3

**Published:** 2017-10-30

**Authors:** Anthony Juritsch, Yi-Ting Tsai, Mulchand S. Patel, Todd C. Rideout

**Affiliations:** 1 Departments of Exercise and Nutrition Sciences, School of Public Health and Health Professions, University at Buffalo, Buffalo, NY 14214 USA; 2 Biochemistry, Jacobs School of Medicine and Biomedical Sciences, University at Buffalo, Buffalo, NY 14214 USA

**Keywords:** Maternal hypercholesterolemia, Offspring, Phytosterols, Liver, Intestine, mRNA

## Abstract

**Objective:**

An excessive rise in blood lipids during pregnancy may promote metabolic dysfunction in adult progeny. We characterized how maternal phytosterol (PS) supplementation affected serum lipids and the expression of lipid-regulatory genes in the intestine and liver of newly-weaned apo-E deficient offspring from dams fed a chow diet supplemented with cholesterol (0.15%, CH) or cholesterol and PS (2%) (CH/PS) throughout pregnancy and lactation.

**Results:**

Serum lipid concentrations and lipoprotein particle numbers were exacerbated in offspring from cholesterol-supplemented mothers but normalized to chow-fed levels in pups exposed to PS through the maternal diet during gestation and lactation. Compared with the CH pups, pups from PS-supplemented mothers demonstrated higher (p < 0.05) expression of the primary intestinal cholesterol transport protein (Niemann-Pick C1-like 1) and the rate-limiting enzyme in hepatic cholesterol synthesis (HMG-CoAr), suggestive of a compensatory response to restore cholesterol balance. Furthermore, pups from PS-supplemented mothers exhibited a coordinated downregulation (p < 0.05) of several genes regulating fatty acid synthesis including PGC1β, SREBP1c, FAS, and ACC compared with the CH group. These results suggest that maternal PS supplementation during hypercholesterolemic pregnancies protects against aberrant lipid responses in newly-weaned offspring and results in differential regulation of cholesterol and lipid regulatory targets within the enterohepatic loop.

## Introduction

Hypercholesterolemia is a considerable public health issue in the United States affecting roughly 30% of the population [1]. Transient elevations in maternal blood lipids during pregnancy are essential for embryogenesis, early organ development, and whole-body fetal growth [2–5]. However, a growing body of literature suggests that excessive maternal hyperlipidemia during pregnancy, termed maternal supraphysiological hypercholesterolemia (MSPH) [6, 7], can adversely program fetal lipid metabolism predisposing offspring to increased cardiovascular disease (CVD) risk as adults by altering hepatic cholesterol metabolism [8, 9] and accelerating the development of arterial fatty streaks [10] and advanced arterial lesions [11]. Napoli et al. [10] reported that aborted fetuses from hypercholesterolemic mothers had significantly more and larger lesions compared with those from mothers with a normal cholesterol range [10]. A follow up autoptic study including 156 children (1–14 years old) suggested that although fetal fatty streaks may regress after birth, arterial lesions develop ‘strikingly’ faster in children whose mothers were hypercholesterolemic during pregnancy versus normocholesterolemic mothers [11].

As the use of statins in expectant mothers and women trying to conceive is contraindicated due to potential teratogenic effects [12], we have conducted studies to examine if phytosterols (PS), plant-based cholesterol-lowering compounds, have utility in the prevention and management MSPH [13, 14]. Results from these studies suggest that newly-weaned offspring from PS-supplemented mothers during gestation and lactation are largely protected against an early dyslipidemic phenotype compared with offspring from hypercholesterolemic mothers. However, the potential molecular mechanisms associated with these lipid responses have yet to be examined. Thus, the primary objective of this study was to characterize alterations in lipid regulatory gene expression within the enterohepatic loop in newly-weaned apoE deficient offspring exposed to PS through the maternal diet during gestation and lactation. We have used the apoE deficient mouse as this model demonstrates a genetic predisposition to hypercholesterolemia which is further exacerbated upon consumption of a high cholesterol diet [15], responds to the cholesterol-lowering actions of phytosterols (unlike wildtype C57BL6/J mice) [16–19], and has been utilized in previous maternal programming studies to examine excessive early cholesterol exposure [20, 21].

## Main text

### Methods

The experimental design has been described previously [14]. In short, twenty-four mature (3-month old) female mice homozygous for disruption of the apoE gene (apoE^−/−KO^, strain B6.129P2-Apoetm1Unc >/J) were purchased from Jackson laboratory. The mice were randomly assigned (n = 8/group) to 1 of 2 commercial nonpurified diets (Teklad 2019 Harlan Laboratories (% energy from protein, 21.4; fat, 19.9; and carbohydrate, 58.7): (i) cholesterol supplemented chow (0.15%, w/w, CH, TD.140285), and (ii) cholesterol (0.15%, w/w) and PS (2%, w/w) supplemented chow (CH/PS, TD.140286; PS sourced from Forbs Medi-Tech Corp, Kearny, NJ). An additional control group of chow-fed animals were included (n = 8) to provide reference values for the normal blood lipid and lipoprotein measurements in newly-weaned apoE deficient offspring but were not used for subsequent gene expression studies. Females were mated for 1 week (1 male per 2 females) with male apoE^−/−KO^ breeders (maintained on a chow diet) and litters were culled to n = 6 pups per dam to minimize variability in postnatal pup development influenced by litter size. Throughout the suckling period the dams remained on their respective diets. At weaning (d21), the dams and pups were anesthetized with isoflurane for blood and tissue collection.

Fasting (15 h) blood was collected by cardiac puncture and intestinal and liver tissue were excised and stored at – 80 °C for further processing. Serum cholesterol panel (total-C, HDL-C, and direct LDL-C) and triglyceride (TG) were analyzed by automated enzymatic kits (Sekisui Diagnostics, Lexington, MA, USA) on an ABX Pentra 400 autoanalyzer (Horiba Instruments Inc., Irvine CA, USA) using appropriate calibrators and controls as specified by the manufacturer. Lipoprotein particle number was analysed by nuclear magnetic resonance spectroscopy (LabCorp) [22]. Serum PCSK9 concentration was measured in serum by ELISA according to the manufacturer instructions (R&D Systems, MPC900).

RNA extraction and real-time RT-PCR were conducted according to previously published procedures [13]. Gene expression was analyzed using the 2(-delta delta Ct) method [36]. Sequences of gene primers were based on previously published reports for β-actin [23], peroxisome proliferator-activated receptor α (PPARα) [24], carnitine palmitoyltransferase Iα (CPT1α) [25], peroxisome proliferator-activated receptor gamma coactivator 1-beta (PGC1β) [26], sterol regulatory element binding protein 1c (SREBP1c) [27], acetyl-coA carboxylase 1 (ACC1) [28], fatty acid synthase (FAS) [29], 3-hydroxy-3-methyl-glutaryl-coenzyme A reductase (HMG-CoAr) [30], SREBP cleavage activating protein (SCAP) [30], low-density lipoprotein receptor (LDLr) [31], proprotein convertase subtilisin/kexin type 9 (PCSK9) [32], sterol regulatory element-binding protein 2 (SREBP2) [31], liver X receptor (LXR) [27], ATP binding cassette subfamily A member 1 (ABCA1) [31], ATP binding cassette subfamily G member 1 (ABCG1) [31], ATP binding cassette subfamily G member 5 (ABCG5) [31], ATP binding cassette subfamily G member 8 (ABCG8) [31], niemann-pick C1-like 1 (NPC1L1) [33], organic solute transporter α and β (Ostα/β) [34], farnesoid X-activated receptor (FXR) [31], microsomal triglyceride transfer protein (MTP) [31], fatty acid binding protein 2 (FABP2) [35], cluster of differentiation 36 (CD36) [36], and low-density lipoprotein receptor (LDLR) [37].

### Statistical analyses

Litters from each dam were counted as a single observation. Blood lipids and lipoproteins concentrations in the main treatment groups (CH and CH/PS) were compared alongside normal chow-fed offspring using a general linear model ANOVA with a Bonferonni post hoc test [38]. Gene expression patterns were compared between the CH and CH/PS groups only using a one-way ANOVA. Data were analyzed with SPSS 16 for Mac (SPSS Inc, Chicago, IL). Data are presented as mean ± SEM. Differences were considered significant at p ≤ 0.05. One dam in the CH/PS group was terminated early, therefore the final number of animals per group was n = 8 chow, n = 8 CH, and n = 7 CH/PS.

### Results

At gestation week 2, cholesterol-fed mothers (CH) demonstrated higher (p < 0.05) serum total cholesterol (+ 70%) and triglyceride (+ 28%) concentrations compared with chow mothers, but this response was normalized in the CH/PS mice (Table 1). Maternal cholesterol feeding during gestation and lactation resulted in dyslipidemic newly-weaned pups with higher (p < 0.05) serum total-cholesterol, LDL-C, and TG compared to chow pups (Table 1). However, maternal PS supplementation protected against this dyslipidemic response with offspring from PS-supplemented mothers demonstrating lower (p < 0.05) serum lipids (total-C, LDL-C and TG) compared with the CH groups. No change (p > 0.05) was observed in HDL-C concentrations between the groups. Additionally, offspring from cholesterol-fed mothers (CH) had higher total number of serum LDL, HDL, and VLDL particles compared to the chow group but this was normalized to chow-fed levels upon maternal PS supplementation.

**Table 1:** Serum lipids in dams (gestation week 2) and newly weaned offspring (postnatal day 21) from mothers fed a chow diet, the chow diet supplemented with cholesterol (CH), or cholesterol and phytosterol (CH/PS) during gestation and lactation

Endpoint	Chow	CH	CH/PS
Maternal lipids (mmol/L), gestation week 2			
Total-C	3.73 ± 0.51^a^	6.36 ± 1.23^b^	4.23 ± 0.34^a^
Triglycerides	0.61 ± 0.02^a^	0.78 ± 0.09^b^	0.60 ± 0.02^a^
Offspring lipids (mmol/L), postnatal day 21			
Total-C	10.9 ± 0.4^a^	12.5 ± 0.5^b^	9.4 ± 0.3^c^
LDL-C	2.6 ± 0.1^a^	3.62 ± 0.1^b^	1.88 ± 0.1^c^
HDL-C	0.60 ± 0.0^a^	0.67 ± 0.1^a^	0.53 ± 0.0^a^
Triglycerides	1.13 ± 0.0^a^	2.09 ± 0.4^b^	1.22 ± 0.1^a^
Offspring lipoprotein particle number (µmol/L), postnatal day 21			
Total LDL Particles	378.1 ± 27.7^a^	676.3 ± 114.0^b^	442.5 ± 28.5^a^
Total HDL Particles	12.3 ± 0.5^a^	16.3 ± 1.6^b^	11.5 ± 0.5^a^
Total VLDL Particles	217.9 ± 9.9^a^	292.4 ± 9.2^b^	201.3 ± 6.7^a^

^a, b, c^Groups not sharing a superscript are significantly different (*p* < 0.05). Data are mean ± SE; n = 8 chow, n = 8 CH, n = 7 CH/PS

**Fig. 1:**
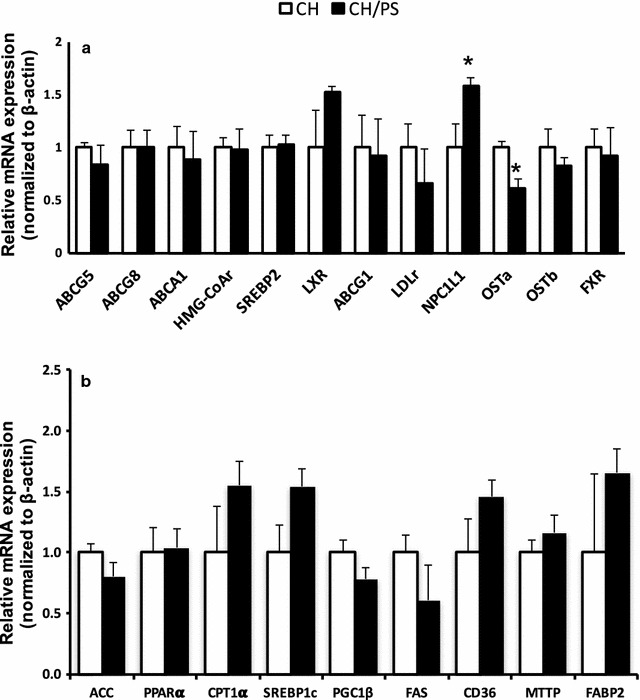
Intestinal mRNA expression of cholesterol (**a**) and lipid regulatory targets (**b**) in newly-weaned apo-E deficient mice from mothers fed a cholesterol-enriched chow diet (CH) or the chow diet supplemented with cholesterol and phytosterol (CH/PS) during gestation and lactation. All genes were normalized to the CH group and expressed relative to β-actin. ^*^ denotes significance (*p* < 0.05). Data are mean ± SE; n = 8 CH, n = 7 CH/PS

Compared with CH offspring, offspring from PS-supplemented mothers demonstrated higher (*p* < 0.05) intestinal NPC1L1 (+ 1.6-fold of CH, Fig. 1a) expression and lower (p < 0.05) expression of the alpha sub-unit (OSTa) of the heterodimeric ileal basolateral bile acid transport protein OSTa/OSTb (0.6-fold of CH, Fig. 1a). No changes (p > 0.05) in lipid regulatory targets were observed between the two groups (Fig. 1b).

**Fig. 2:**
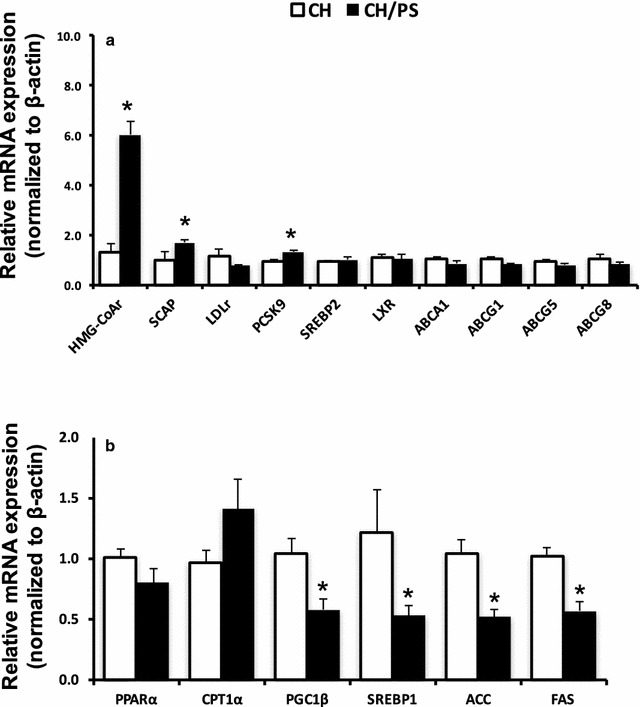
Hepatic mRNA expression of cholesterol (**a**) and lipid regulatory targets (**b**) in newly-weaned apo-E deficient mice from mothers fed a cholesterol-enriched chow diet (CH) or the chow diet supplemented with cholesterol and phytosterol (CH/PS) during gestation and lactation. All genes were normalized to the CH group and expressed relative to β-actin. *denotes significance (*p* < 0.05). Data are mean ± SE; n = 8 CH, n = 7 CH/PS

New-weaned pups from PS-supplemented mothers demonstrated higher (p < 0.05) hepatic mRNA expression of HMG-CoAr (+ 5.4-fold of CH), SCAP (+ 1.7-fold of CH), and PCSK9 (+ 1.4-fold of CH) compared with the CH group (Fig. 2a). However, increased hepatic PCSK9 transcription did not reflect in any change (p > 0.05) in serum PCSK9 concentration between the CH and CH/PS groups (10.8 ± 2.2 vs. 12.6 ± 1.5 µg/mL, respectively). Although no difference was observed between the CH and CH/PS groups in the expression of hepatic fat oxidative regulators (CPT1α or PPARα), pups from PS-supplemented mothers demonstrated a reduction (p < 0.05) in several genes that regulate fatty acid synthesis including PGC1β (0.5-fold of CH), SREBP1c (0.43-fold of CH), FAS (0.55-fold of CH), and ACC (0.49-fold of CH) (Fig. 2b).

### Discussion

Serum lipids and lipoprotein concentrations were exacerbated in offspring from cholesterol-supplemented mothers but were normalized to chow-fed levels in pups exposed to PS through the maternal diet during gestation and lactation. Gene expression results indicate that due to a likely interference with in utero and/or postnatal cholesterol transfer, offspring from PS supplemented mothers had enhanced intestinal cholesterol absorption and hepatic cholesterol synthesis as reflected by higher NPC1L1 and HMG-CoAr expression, respectively. Results further suggest that early exposure to PS resulted in a coordinated reduction in hepatic lipogenic gene expression which may underlie the TG-lowering response observed in these animals.

The effects of maternal PS supplementation on lipid metabolism in newly-weaned offspring is likely mediated through multiple contributing mechanisms including the limitation of excessive cholesterol transfer during gestation, the alteration of lipid milk composition during the suckling period, and/or direct effects of PS on lipid-regulatory gene expression patterns within offspring. The expression of a variety of lipid transport proteins in placental trophoblasts and endothelial cells regulates the transfer of lipids and cholesterol from the maternal to the fetal circulation [39, 40]. A lowering of total body cholesterol balance in PS-supplemented mothers, as evidenced by reduced gestational serum cholesterol and TG, would likely limit cholesterol transfer between the mother and developing fetus. However, as far as we are aware, there are no studies examining placental lipid transport in response to PS. It is equally likely that interruption of maternal lipid transfer during lactation could have contributed to the observed lipid changes in offspring through altered milk composition of cholesterol and/or TGs. However, in a previous human study, Mellies et al. [41] detected no change in cholesterol concentration in breast milk following maternal PS supplementation in the lactation period (30 days) despite a reduction in maternal plasma cholesterol levels [41]. Whether maternal PS supplementation inhibited cholesterol transfer to offspring in utero or during lactation, gene expression data in both the intestine and liver support a compensatory increase in intestinal cholesterol uptake through the primary intestinal cholesterol transport protein NPC1L1 [42] and the rate-limiting enzyme in hepatic cholesterol, HMG-CoAr. This gene expression pattern may well reflect an effort to restore cholesterol lipid balance as cholesterol is critical for early development [43]. Although there are no other maternal PS supplementation studies with which to compare our gene expression data, previous PS supplementation studies in adult animals have reported variable expression of NCP1L1 [44–47] and a more consistent increase in HMG-CoAr expression [48, 49] and cholesterol synthesis [50–52].

We observed a coordinated reduction in the hepatic expression of a host of regulatory genes involved in de novo fatty acid synthesis including the rate-limiting enzymes ACC and FAS a reduction in the expression of PGC1β and SREBP1, critical molecular regulators that enhance hepatic fat synthesis [53]. In support of these observations, we recently reported a reduction in de novo lipogenesis and an associated down-regulation of hepatic FAS protein abundance in adult male Syrian golden hamsters fed a high fat diet supplemented with PS [54]. It is tempting to speculate that the reduction in hepatic lipogenic genes may be related to the lower serum TG and VLDL particles observed in pups from PS-supplemented mothers. In support of a TG-lowering mechanism of hepatic origin, Schonewille et al. recently reported a reduction in hepatic VLDL secretion in male C57BL/6 J mice fed a high fat diet supplemented with 3.1% PS or stanol esters for 3 weeks [55].

In summary, maternal hypercholesterolemia during pregnancy resulted in an overt dyslipidemia in newly-weaned pups that was normalized through maternal PS supplementation throughout the pregnancy and lactation periods. Pups from PS-supplemented mothers demonstrated higher intestinal NPC1L1 and hepatic HMG-CoAr mRNA expression, suggestive of a compensatory response to restore cholesterol balance. The effects of maternal PS supplementation in normalizing blood TG concentration and VLDL particle numbers is likely associated with a coordinated down-regulation of hepatic lipogenic gene expression.

## Limitations

Although our data demonstrates hepatic transcriptional changes in lipid regulatory genes in newly-weaned offspring exposed to excessive cholesterol and the protective effects of maternal PS supplementation, it is unclear if these changes are the result of prenatal versus postnatal exposure and if the observed hepatic adaptations will persist into adult life.

## Abbreviations

PS: phytosterols
CH: cholesterol supplemented chow
CH/PS: cholesterol supplemented chow with PS
PPARα: peroxisome proliferator-activated receptor α
CPT1α: carnitine palmitoyltransferase Iα
PGC1β: peroxisome proliferator-activated receptor gamma coactivator 1-beta
SREBP1c: sterol regulatory element binding protein 1c
ACC1: acetyl-coA carboxylase 1
FAS: fatty acid synthase
HMG-CoAr: 3-hydroxy-3-methyl-glutaryl-coenzyme A reductase
SCAP: SREBP cleavage activating protein
LDLr: low-density lipoprotein receptor
PCSK9: proprotein convertase subtilisin/kexin type 9
SREBP2: sterol regulatory element-binding protein 2
LXR: liver X receptor
ABCA1: ATP binding cassette subfamily A member 1
ABCG1: ATP binding cassette subfamily G member 1
ABCG5: ATP binding cassette subfamily G member 5
ABCG8: ATP binding cassette subfamily G member 8
NPC1L1: niemann-pick C1-like 1
Ostα/β: organic solute transporter α and β
FXR: farnesoid X-activated receptor
MTP: microsomal triglyceride transfer protein
FABP2: fatty acid binding protein 2
CD36: cluster of differentiation 36
LDLR: low-density lipoprotein receptor
